# Effects of resistance training with controlled versus self-selected repetition duration on muscle mass and strength in untrained men

**DOI:** 10.7717/peerj.8697

**Published:** 2020-03-06

**Authors:** Talisson Santos Chaves, Thaís Marina Pires de Campos Biazon, Lucas Marcelino Eder dos Santos, Cleiton Augusto Libardi

**Affiliations:** 1 MUSCULAB-Laboratory of Neuromuscular Adaptations to Resistance Training, Department of Physical Education, Federal University of São Carlos–UFSCar, São Carlos, São Paulo, Brazil; 2 Laboratory of Cardiopulmonary Physiotherapy-LACAP, Department of Physiotherapy, Federal University of São Carlos–UFSCar, São Carlos, São Paulo, Brazil

**Keywords:** Strength training, Repetition velocity, Cadence, Muscle hypertrophy, Muscle strength

## Abstract

The aim of this study was to compare the effect of self-selected repetition duration (SELF), with and without volume load (VL) equalized with controlled repetition duration (CON) on muscle strength and hypertrophy in untrained males. We used a within-subjects design in which 20 volunteers (age: 24.7 ± 2.9 years) had one leg randomly assigned to CON (i.e., 2 s concentric, 2 s eccentric) and the other to SELF or to self-selected repetition duration with equalized volume load (SELF-EV). One repetition maximum (1-RM) and muscle cross-sectional area (CSA) were measured at baseline (Pre) and after (Post) resistance training (RT; 2×/wk for 8 weeks). For the main study variables (1-RM and muscle CSA), a mixed-model analysis was performed, assuming repetition duration (SELF, SELF-EV and CON), and time (Pre and Post) as fixed factors and the subjects as random factor for each dependent variable (1-RM and CSA). All RT protocols showed significant increases in values of 1-RM from Pre (CON: 73.7 ± 17.6 kg; SELF: 75.9 ± 17.7 kg; and SELF-EV: 72.6 ± 16.9 kg) to Post (CON: 83.4 ± 19.9 kg, effect size (ES): 0.47; SELF: 84 ± 19.1 kg, ES: 0.43; and SELF-EV: 83.2 ± 19.9 kg, ES: 0.57, *P* < 0.0001). Muscle CSA values increased for all protocols from Pre (CON: 12.09 ± 3.14 cm^2^; SELF: 11.91 ± 3.71 cm^2^; and SELF-EV: 11.93 ± 2.32 cm^2^) to Post (CON: 13.03 ± 3.25 cm^2^, ES: 0.29; SELF: 13.2 ± 4.16 cm^2^, ES: 0.32; and SELF-EV: 13.2 ± 2.35 cm^2^, ES: 0.53, *P* < 0.0001). No significant differences between protocols were found for both 1-RM and CSA (*P* > 0.05). Performing RT with SELF, regardless of VL, was equally effective in inducing increases in muscle strength and hypertrophy compared to CON in untrained men.

## Introduction

In order to increase muscle strength and mass (i.e., muscle hypertrophy), the manipulation of resistance training (RT) variables such as volume (i.e., sets × repetitions), intensity, exercise type, weekly frequency, rest, muscle action (i.e., concentric vs. eccentric) and repetition duration (RD) (i.e., time under tension (TUT) per repetition) has been recommended (ACSM, 2009, 2011; Kraemer & Ratamess, 2004). Concerning RD, the ACSM (2009) recommends a moderate duration (e.g., 2 s for concentric action and 2 s for eccentric action (2 s: 2 s)) for untrained. However, several studies have neglected RD control by not reporting or simply not monitoring this variable (Angleri, Ugrinowitsch & Libardi, 2017; Barcelos et al., 2018; De Salles et al., 2016; Lasevicius et al., 2018; Nobrega et al., 2018b; Radaelli et al., 2015). In this sense, it is unknown whether the lack of control of this variable (i.e., RD self-selected) would have any impact on RT adaptations.

Although to date no study has investigated the chronic effects of self-selected RD (SELF) with controlled RD (CON) on muscle strength and mass, a study from our laboratory analyzing the acute effects of RD manipulation on a RT session showed that a protocol with SELF (i.e., on average 1.5 s: 1.5 s) resulted in higher electromyography (EMG) amplitude compared with CON (Nobrega et al., 2018a). In fact, changes in RD seem to influence the amplitude of EMG, with shorter RDs resulting in larger EMG amplitudes (Sakamoto & Sinclair, 2012). However, muscle fiber activation and subsequent anabolic signaling are independent of EMG amplitude when resistance exercise is performed to concentric muscle failure (Morton et al., 2019). Additionally, similar muscle strength gains and hypertrophy between RT protocols with different EMG amplitudes has been recently shown (Mitchell et al., 2012; Morton et al., 2016; Nobrega et al., 2018b; Schoenfeld et al., 2014). Thus, although suggestive, a higher EMG amplitude is not necessarily an advantage for a SELF protocol. Other acute studies have also shown that SELF and CON can affect resistance exercise volume and TUT per repetition (LaChance & Hortobagyi, 1994; Nobrega et al., 2018a). Recently, Nobrega et al. (2018a) showed that the volume (e.g., number of repetitions or volume load (sets × repetitions × load)) is higher in SELF compared to CON. On the other hand, the TUT per repetition was higher in CON compared with SELF (2 s: 2 s vs. 1.5 s: 1.5 s, respectively). In fact, both volume and TUT may influence muscle anabolic response (Burd et al., 2012, 2010). However, a recent meta-analysis has shown that when RT is performed to concentric muscle failure, the increases in muscle strength and mass are similar if training is performed with RD ranging from 0.5 to 8 s (Schoenfeld, Ogborn & Krieger, 2015; Davies et al., 2017), which comprises the TUT range of both protocols (i.e., SELF and CON). On the other hand, meta-analyzes showed that higher volume (i.e., number of sets, which directly influences the number of repetitions in the session and VL) produced greater increases in muscle strength (Ralston et al., 2017) and hypertrophy (Krieger, 2010; Schoenfeld, Ogborn & Krieger, 2017). Thus, it is reasonable to speculate that SELF may promote greater muscle strength gains and hypertrophy than CON, which may not happen when the VL (which is considered the most appropriate method for volume quantification when comparing protocols (Schoenfeld & Grgic, 2018)) of SELF is equalized to CON. However, this hypothesis has not been previously tested.

Thus, the aim of the study was to compare the effect of SELF, with and without VL equalized with CON, on muscle strength and hypertrophy. We hypothesized that SELF would produce greater VL, thus resulting in larger muscle strength and hypertrophy gains than CON protocol. In addition, we hypothesized that there would be no difference between SELF with equalized VL (SELF-EV) and CON.

## Methods

### Participants

Twenty healthy untrained men (age: 24.7 ± 2.9 years, height: 176 ± 7.4 cm, body mass: 76.2 ± 10.5 kg) were recruited for this study. As inclusion criteria for participation, subjects were not engaged in resistance training within the last 6 months and had no functional limitations (e.g., due to joint or muscle injuries) for performing the one repetition maximum test (1-RM) or training protocols. Prior to the study initiation all participants were informed of the procedures, risk and benefits, and were asked to provide written informed consent. All procedures performed in the study were in accordance with the ethical standards of the institutional research committee and with the 1964 Helsinki declaration. The Federal University of São Carlos (UFSCar-5504) granted ethical approval to carry out the study within its facilities (approval number: 65677217.6.0000.5504).

### Study design

Initially, the participants visited the laboratory to answer the physical activity readiness questionnaire (PAR-Q) and to be familiarized with the 1-RM test. After 72 h, the participants performed the unilateral 1-RM test in the leg extension exercise on both legs, and it was necessary to perform one more test if there was a load variation greater than 5% between the tests performed (Levinger et al., 2009). After 72 h from the 1-RM test, the cross-sectional area of the vastus lateralis muscle (CSA) was assessed by ultrasound. To reduce inter-subjects variability an intra-subject design was used in which each participant’s leg was allocated in a randomized and balanced way to either: (1) Control RD (CON); (2) Self-selected RD with equalized volume load (SELF-EV); Self-selected RD without equalized volume load (SELF). The CON group was defined as a positive control for all of the participants. Thus, twenty legs were allocated to the CON protocol (10 dominant and 10 non-dominant legs). Contralateral legs were then allocated in a balanced way to either SELF (*n* = 10) or SELF-EV protocol (*n* = 10), according to 1-RM values. Following muscle CSA evaluations, the training period was started (2×/wk for 8 weeks). After four weeks of training, 1-RM was reassessed unilaterally 72 h after the 8th training session, to readjust the load. The training continued for another 4 weeks with the adjusted load. Muscle CSA was assessed 72 h after the last RT session, with subsequent assessments of final 1-RM test.

### Maximal dynamic strength test

Maximum dynamic strength was evaluated unilaterally via 1-RM leg extension on both legs by following the recommendations of Brown & Weir (2001). Initially, participants performed a general warm up on a cycle ergometer for 5 min, followed by two sets of specific warm-up. The first and second sets consisted of eight repetitions with 50% of the estimated 1-RM, and three repetitions with 70% of the estimated 1-RM, respectively, with a 2 min rest between warm-up sets. After the warm-up, the 1-RM was obtained through the greatest load lifted between 3 and 5 attempts, respecting a 3 min interval between attempts. Data collection and analysis were performed by the same evaluator at all time points. The coefficient of variation and typical error of 1.03% and 0.7 kg were obtained from two repeated measures with 72 h intervals between testing sessions.

### Muscle cross-sectional area

Data were collected as previously described in Lixandrão et al. (2014). Specifically, participants were instructed to refrain from strenuous physical activities for at least 72 h before each CSA assessment (Damas et al., 2016; Newton et al., 2008). Previously to the acquisition of images, participants were advised to lay in a supine position for 20 min to ensure fluid redistribution. The images were collected through a B-mode US with a linear probe set at 7.5 MHz (MySono U6; Samsung, São Paulo, Brazil). Transmission gel was applied in the area where the images were acquired, ensuring acoustic coupling, without compressing the epidermis. The midpoint between the greater trochanter and the lateral epicondyle of the femur was used for the acquisition of CSA images. Images were acquired in the sagittal plane. To guide the displacement of the probe, the skin was transversely marked at intervals of 2 cm. Sequential images of the vastus lateralis muscle started at the point of alignment of the upper edge of the probe with the most medial skin mark (over the rectus femoris muscle) and ended at the lateral aspect of the thigh. Images were recorded every 2 cm and Power Point was used to open the sequence of images (Microsoft, Redmond, WA, USA), which were manually rotated to reconstruct the entire fascia of vastus lateralis muscle, and saved as a new image. Figure files were opened in the ImageJ software and the “polygonal” function was used to determine vastus lateralis CSA. ImageJ “polygonal” functional was calibrated using a known distance marked in the US unit. Importantly, data collection and analysis were performed by the same evaluator at all time points. The coefficient of variation and typical error of 1.37% and 0.14 cm^2^ were obtained from two repeated measures separated by a 72 h interval.

### Resistance exercise protocols

All RT protocols were performed unilaterally using a conventional leg-extension machine, twice a week for 8 weeks (16 training sessions). At the beginning of each RT session, participants performed a general warm-up on a cycle ergometer (Ergo-Fit^®^; Pirmasens, Rheinland-Pfalz, Germany) pedaling at 20 km·h^−1^ for 5 min. For the SELF and CON protocols, the participants performed 3 sets to concentric muscle failure at 70% 1-RM with 2 min of rest between sets for both protocols. A 2 min interval was used between the end of the control leg session and the beginning of the contralateral leg. During SELF-EV, the same intensity and between-sets rest intervals were utilized, however, the number of sets varied (2–3 sets) due to VL equalization with the CON protocol. To ensure load progression, the intensity was readjusted at the end of the 4th week (eighth session). The RD in CON was controlled using a metronome, with both concentric and eccentric actions lasting 2 s (2 s: 2 s). For SELF and SELF-EV protocols, the RD was determined voluntarily by the individual, with no research intervention. To calculate the mean TUT per repetition in SELF and SELF-EV, the set time (in seconds) was recorded, using a stopwatch, and divided by the number of repetitions performed in the set. All procedures were performed by the same evaluator. Sets were carried out to the point of either concentric muscle failure (i.e., inability to perform another concentric repetition while maintaining proper form) or not been able to maintain proper repetition duration (only for CON protocol).

### Volume load equalization

As a result of our experimental design, VL was equalized between the SELF-EV and CON protocols. For this, the CON protocol was performed first, and after the end of the exercise, the VL (i.e., repetitions × sets × load) was calculated. Then, the calculated VL in the CON leg was used as the reference VL value that should be achieved in the contralateral leg (SELF-EV), which was subsequently trained. For this, we divided the VL of the CON protocol by the intensity used for the SELF-EV protocol, which was previously set at 70% 1-RM. Thus, we defined the number of repetitions that the SELF-EV protocol should perform to obtain the same VL as the CON group. It is important to emphasize that even though prior sets were performed until concentric muscle failure, the last set was interrupted as soon as VL reached the calculated value. Thus, muscle failure was not a necessary condition on SELF-EV’s final set.

### Statistical analysis

After a visual inspection of the data, the Gaussian distribution was observed by Shapiro–Wilk test, with non-normal data log-transformed prior to statistical analysis. A one-way ANOVA of repeated measures was used to compare the 8-week accumulated VL and number of repetitions between SELF, SELF-EV and CON protocols. The TUT per repetition was not normally distributed and data transformation was not possible. Thus, to analyze this variable we used the non-parametric Kruskal–Wallis test. For the main study variables (1-RM and muscle CSA) a mixed-model analysis was performed, assuming RD (SELF, SELF-EV and CON) and time (Pre and Post) as fixed factors and the subjects as random factor for each dependent variable (1-RM and CSA). As a result of our experimental design, 20 legs were allocated to CON, while SELF and SELF-EV remained with 10 legs each. Thus, 10 simulations in which 10 legs were randomly removed from the CON condition were analyzed in order to test if different statistical results would be found when all 20 legs were considered vs. when only 10 legs were considered. The data was statistically analyzed using *n* = 20 for CON, *n* = 10 for SELF and *n* = 10 for SELF-EV, once there were no statistical differences between simulations for any dependent variable. In case of a significant *F* value, Tukey’s adjustments were performed for multiple comparisons. *P* values < 0.05 were considered significant. Effect sizes (ES) were calculated for 1-RM and CSA using the changes from Pre to Post. ES were classified as “small” if lower than 0.2, “medium” if between 0.2 and 0.5, and “large” if higher than 0.8 (Cohen, 1988).

## Results

### Time under tension per repetition, number of repetitions per session and volume load

Time under tension per repetition was higher for the CON protocol (4.0 ± 0 s) compared to SELF (1.8 ± 0.3 s) and SELF-EV (1.7 ± 0.4 s) (*H* = 33.64, *P* < 0.001). The number of repetitions per session was greater in SELF (35.20 ± 6.39 repetitions) protocol compared with CON (23.95 ± 3.91 repetitions) and SELF-EV (24.50 ± 3.80 repetitions) (*F*_(2, 37)_ = 20.70, *P* < 0.001). Consequently, the VL accumulated in 8 weeks was higher for the SELF protocol (31,425 ± 11,717 kg) compared to CON (20,401 ± 5,939 kg) and SELF-EV (19,512 ± 4,612 kg) (*F*_(2, 37)_ = 8.524, *P* < 0.001) (Table 1).

**Table 1:** Time under tension (TUT) per repetition, number of repetitions per session and volume load.

Variables	CON	SELF-EV	SELF
TUT per repetition (s)	4.0 ± 0*	1.7 ± 0.4	1.8 ± 0.3
Number of repetitions per session	23.9 ± 3.9	24.5 ± 3.8	35.2 ± 6.4^†^
Volume load (kg)	20,401.1 ± 5,939.7	19,512.6 ± 4,612.6	31,425.1 ± 11,717.7^†^

**Notes:**

* Significant difference compared to SELF-EV and SELF (*P* < 0.05).

† Significant difference compared to CON and SELF-EV (*P* < 0.05).

### Maximal dynamic strength (1-RM)

**Figure 1:**
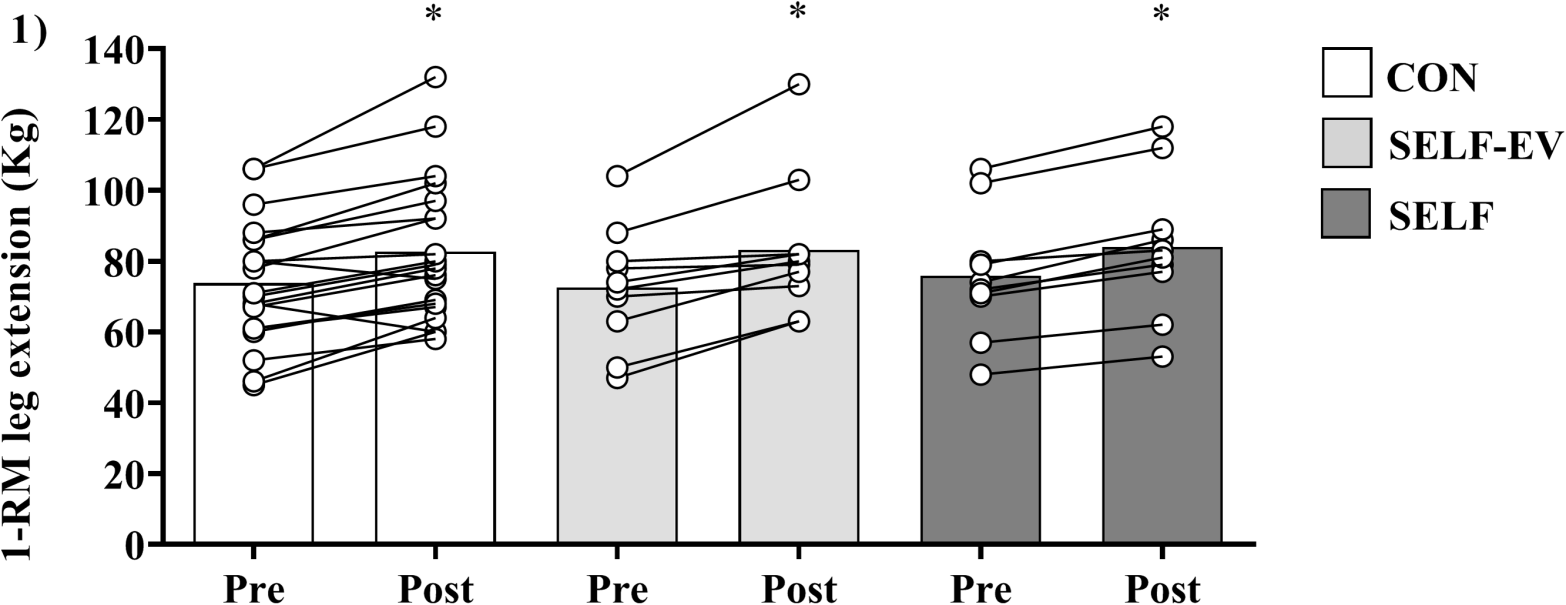
Increases in maximal dynamic strength at 8 weeks. Maximal dynamic strength (1-RM) at baseline (Pre) and after 8 weeks (Post) of resistance training with controlled repetition duration (CON), resistance training with self-selected repetition duration and equalized volume (SELF-EV) and resistance training with self-selected repetition duration (SELF). Results are presented as means ± SD. *Significant difference compared to Pre (main time effect, *P* < 0.0001).

All training protocols showed significant increases in values of 1-RM from Pre (CON: 73.7 ± 17.6 kg; SELF: 75.9 ± 17.7 kg; and SELF-EV: 72.6 ± 16.9 kg) to Post (CON: 83.4 ± 19.9 kg, ES: 0.47 (medium); SELF = 84 ± 19.1 kg, ES: 0.43 (medium); and SELF-EV: 83.2 ± 19.9 kg, ES: 0.57 (medium); main effect of time, *F*_(1, 37)_ = 64.38, *P* < 0.001). No significant main effect of group (*F*_(2, 37)_ = 0.04, *P* = 0.96) or groups vs. time interaction (*F*_(2, 37)_ = 0.48, *P* = 0.62) were found (Fig. 1).

### Muscle cross-sectional area

**Figure 2:**
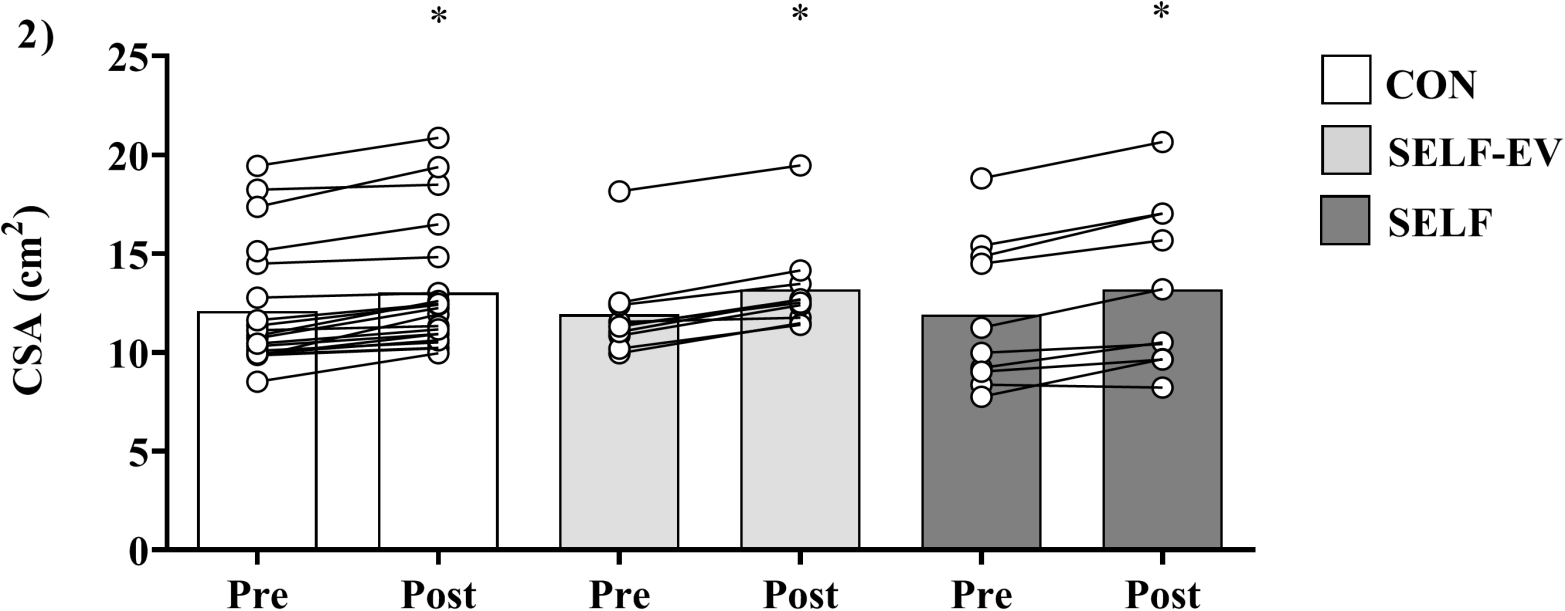
Increases in muscle cross-sectional area at 8 weeks. Muscle cross-sectional area (CSA) before (Pre), and after 8 weeks (Post) of resistance training with controlled repetition duration (CON), resistance training with self-selected repetition duration and equalized volume (SELF-EV) and resistance training with self-selected repetition duration (SELF). Results are presented as means ± SD. *Significant difference compared to Pre (main time effect, *P* < 0.0001).

Vastus lateralis muscle CSA values increased for all training protocols from Pre (CON = 12.09 ± 3.14 cm^2^; SELF = 11.91 ± 3.71 cm^2^; and SELF-EV = 11.93 ± 2.32 cm^2^) to Post (CON = 13.03 ± 3.25 cm^2^, ES: 0.29 (medium); SELF = 13.2 ± 4.16 cm^2^, ES: 0.32 (medium); and SELF-EV = 13.2 ± 2.35 cm^2^, ES: 0.53 (medium); main effect of time, *F*_(1, 37)_ = 127.05, *P* < 0.001). No significant main effect of group (*F*_(2, 37)_ = 0.00, *P* = 1.00) or groups vs. time interaction (*F*_(2, 37)_ = 1.49, *P* = 0.23) were found (Fig. 2).

## Discussion

To the authors’ knowledge, this is the first study that compared the effects of controlled (CON) and self-selected (SELF) RD on muscle strength and mass. Our main findings showed that SELF is equally effective in promoting increases in strength and muscle mass compared to CON, regardless of the VL produced by these protocols.

In our study, we observed increases between 10–14% in maximal strength and 7–10% in muscle hypertrophy after 8 weeks, which is in line with a previous study also performed with untrained subjects (Ahtiainen et al., 2016). Additionally, we demonstrated that SELF results in greater number of repetitions per session and VL, which was also observed in a previous study from our laboratory (Nobrega et al., 2018a). Nevertheless, the increase in muscle strength and hypertrophy was similar to the RT protocols with lower volume (CON and SELF-EV), contradicting our initial hypothesis. In fact, recent meta-analyses seem to be in contrast with the ones reported herein (Krieger, 2010; Ralston et al., 2017; Schoenfeld, Ogborn & Krieger, 2017). The authors showed that higher RT volumes (number of sets, which directly influence the number of repetitions in the session and VL) produced greater increases in muscle strength (Ralston et al., 2017) and hypertrophy (Krieger, 2010; Schoenfeld, Ogborn & Krieger, 2017). On the other hand, previous studies have suggested that there may be a plateau and possibly even a decline in adaptations (i.e., muscle strength and hypertrophy) after a certain volume (number of repetitions or sets) of RT (Barbalho et al., 2019; Wernbom, Augustsson & Thomee, 2007). Additionally, as in the current study, others studies have also shown similar increases in strength gains and muscle hypertrophy despite differences in VL (Barcelos et al., 2018; Bottaro et al., 2011; Mitchell et al., 2012; Nobrega et al., 2018b; Ostrowski et al., 1997). Thus, our findings suggest that there may be a “ceiling effect” to the dose-response relationship between RT volume and increases in muscle strength and hypertrophy.

Although RD control is suggested during RT (ACSM, 2009), our results show that the absence of control in our study did not affect gains in strength and muscle mass. In fact, the differences in TUT per repetition between protocols (CON: 4 s (moderate RD); SELF-EV: 1.7 s (fast RD) and SELF: 1.8 s (fast RD)), are within an RD range capable of maximizing muscle strength and hypertrophy (i.e., fast/moderate–slow (0.5 to 8 s)) (Davies et al., 2017; Schoenfeld, Ogborn & Krieger, 2015). In addition, it is important to note that most studies included in the meta-analyses (Schoenfeld, Ogborn & Krieger, 2015; Davies et al., 2017) performed RT protocols up to concentric muscle failure. This suggests that when the stimulus is performed to failure, RD manipulation may not be relevant to muscle strength and mass gains. Although these findings highlight the importance of concentric muscle failure, our findings suggest that when exercise is performed at moderate load (e.g., 70% 1-RM), RD manipulation may not be necessary even when the exercise is stopped before concentric muscle failure. In our study, only the SELF protocol was performed to concentric muscle failure in all sets and possibly was the only one that experienced the maximum level of fatigue during RT. In fact, this can be evidenced due to the intra-subject design of the present study, which enabled the analysis of the difference in the number of repetitions per set in the SELF (first set: 14.4 ± 3.2, second set: 11.3 ± 1.8 and third set: 9.4 ± 2.0 repetitions) compared to CON (first set: 9.7 ± 2.4, second set: 7.8 ± 1.2 and third set: 6.6 ± 0.7 repetitions) protocols. However, despite this difference, RT adaptations were not influenced. This is in accordance with a recent study by our laboratory, which demonstrated that RT protocols performed up to or near concentric muscle failure (i.e., volitional interruption) produce similar gains in muscle strength and hypertrophy (Nobrega et al., 2018b). Altogether, these findings indicated that if a set is performed until, or close to, concentric muscle failure, the exercise can be performed without controlling the RD, conferring greater autonomy to individuals during the RT practice.

This study is not without limitations. First: The cross-education effect is an important point to be discussed in an intra-subject study design. We consider that the effect of training in our study was greater than the effect of cross-education on muscle strength (Carroll et al., 2006). Considering that both legs performed resistance training, any cross-education effect on muscle strength would have occurred for both sides. Furthermore, we consider that the cross-education has less effect on muscle strength and hypertrophy than the biological variability (between-subject design). In this sense, as between-leg responses are affected by biological variability, an intra-subject design seem to be very effective in controlling such variability. Second: Only untrained subjects were included in the experiment, restricting our conclusions to this population. Although we cannot extrapolate such results to trained individuals, a body of literature demonstrates that, for trained individuals, manipulating one single RT variable at a time results in similar increases in strength and muscle hypertrophic responses to RT when each exercise set is performed until, or close to, concentric failure (Ahtiainen et al., 2005; Morton et al., 2016; Nobrega et al., 2018b; Schoenfeld et al., 2016). Moreover, a previous study showed that intrinsic individual factors (e.g., biological individuality) of trained subjects are key determinants of the changes in muscle CSA compared to extrinsic manipulation of common RT variables (Damas et al., 2019). Thus, we speculate that the findings of our study would not be different in trained subjects. Such hypothesis needs investigation. Third: In our study, we did not control the diet or make a dietary recall. However, the participants were asked to maintain the same eating habits. In addition, intra-design may minimize possible differences in macronutrient intake between subjects. Fourth: We did not consider previous imbalances between legs. However, we were careful not to include participants any joint or muscle injuries. Such criteria may have minimized possible differences between members. In addition, there were no differences in strength and muscle mass baseline mean values between groups. Considering that an intra-subject design was used, any substantial muscle imbalance between legs would have impacted the groups’ mean. Fifth: Unfortunately, the TUT per repetition throughout the set was not controlled. However, a study from our laboratory used an electrogoniometer to check the variation of TUT within the same set during SELF and CON protocols (Nobrega et al., 2018a). The results showed that TUT per repetition in the SELF protocol increased throughout the sets, possibly due to accumulated fatigue. Thus, it is possible that the same occurred in the present study. Finally, only a single joint exercise was used in the study, which may have negatively impacted the ecological validity of the study.

## Conclusions

In conclusion, our results show that it is not necessary to control RD when the goal is to increase maximal strength and muscular hypertrophy in untrained men. Additionally, despite the decrease in VL, RD control does not affect such adaptations. Therefore, trainers and coaches can choose the strategy they prefer when the aim of resistance training is to increase strength and muscle mass.

## Supplemental Information

10.7717/peerj.8697/supp-1 Supplemental Information 1 Raw data related to study dependent variables.

## Additional Information and Declarations

### Data Availability

The following information was supplied regarding data availability: The raw measurements are available in the Supplemental File.
